# Interleukin-33: Friend or Foe in Gastrointestinal Tract Cancers?

**DOI:** 10.3390/cells12111481

**Published:** 2023-05-26

**Authors:** Laura Francesca Pisani, Isabella Teani, Maurizio Vecchi, Luca Pastorelli

**Affiliations:** 1 Gastroenterology and Endoscopy Unit, IRCCS Policlinico San Donato, 20097 San Donato Milanese, Italy; 2 Immunology and Functional Genomics Unit, Centro Cardiologico Monzino, IRCCS, 20138 Milan, Italy; 3 Department of Medicine, University of Verona, 37129 Verona, Italy; isabella.teani@gmail.com; 4 Gastroenterology and Endoscopy Unit, Fondazione IRCCS Ca’ Granda Ospedale Maggiore Policlinico, 20122 Milan, Italy; 5 Department of Pathophysiology and Transplantation, University of Milan, 20122 Milan, Italy; 6 Department of Health Sciences, University of Milan, 20122 Milan, Italy; 7 Gastroenterology and Liver Unit, ASST Santi Paolo e Carlo, 20142 Milan, Italy

**Keywords:** gastric cancer, colorectal cancer, esophageal cancer, interleukin-33, inflammation

## Abstract

Accumulating evidence suggests that Interleukin-33 (IL-33), a member of the IL-1 family, has crucial roles in tissue homeostasis and repair, type 2 immunity, inflammation, and viral infection. IL-33 is a novel contributing factor in tumorigenesis and plays a critical role in regulating angiogenesis and cancer progression in a variety of human cancers. The partially unraveled role of IL-33/ST2 signaling in gastrointestinal tract cancers is being investigated through the analysis of patients’ samples and by studies in murine and rat models. In this review, we discuss the basic biology and mechanisms of release of the IL-33 protein and its involvement in gastrointestinal cancer onset and progression.

## 1. Introduction

Interleukin-33 (IL-33) was first identified in 2003 in hypertrophic veins as a nuclear factor preferentially expressed in high endothelial venules [1]; then, in 2005, IL-33 was recognized as a new member of the IL-1 cytokine family [2]. The binding of IL-33 to its receptor, ST2, otherwise defined as IL-1 receptor-like 1 (IL1RL1) [2,3], is required for its biological activities. Human IL-33 can be subdivided by purpose into three distinct domains: at the N-terminal end (aa 1–65), the region required for nuclear localization and chromatin binding, in the central domain (aa 66–111), a portion enabling the interaction with nuclear factor-B (Nf-kB), and at the region for ST2 binding located in the C-terminal IL-1-like cytokine domain (aa 112–270) [4]. Unlike other IL-1 family members, IL-33 is inactivated through caspase-1 cleavage, while cleavage by cathepsin G and neutrophil elastase enhances its bioactivity [5,6]. IL-33 expression appears to be restricted by cell type in barrier epithelia, suggesting its possible involvement in early immune responses against invasive pathogens [2]. IL-33 is also considered an ‘alarmin’ released upon cellular stress or damage to promote or amplify inflammation [7,8]. Beyond its role in immunity/inflammation, IL-33 appears to exert some effects on cellular proliferation and differentiation; in fact, mice treated with exogenous IL-33 developed a prominent goblet cell hyperplasia in the airways and along the gastrointestinal tract [2].

IL-33 is predominantly present in stromal cells, including fibroblasts, cancer-associated fibroblasts (CAF), pericytes, and mesenchymal stromal cells; furthermore, endothelial and epithelial cells express it, as well as smooth muscle cells and specific populations of hematopoietic cells, such as macrophages [2,9,10]. These various cellular types are a part, together with the extracellular matrix (ECM) and the blood and lymphatic vascular networks, of the tumor microenvironment (TME), a complex and rich multicellular network that can dynamically regulate cancer progression and response to therapies [11]. The ability of these non-neoplastic cells to influence tumor cells and regulate inflammatory microenvironments is conveyed by the plethora of hormones, growth factors, chemokines, and cytokines that they secrete [12]. Among these is IL-33, a potent emerging modulator of the TME through the recruitment of immune cells able to shape tumor phenotype and promote malignancy or impose tumor regression [2,13]. IL-33’s role in tumorigenesis was first identified in breast cancer [14,15], and more recently, omics studies and single-cell sequencing have demonstrated that, upon stimulation by IL-33 and IL-5 as well as CD4+ T cells, eosinophils could enhance CD8+ T-cell activation [16]. Systemic accumulation of eosinophils in tumor cells mediated by IL-33 is associated with improved progression-free and overall survival in triple-negative breast cancer (TNBC) [17].

IL-33 exerts its functions in different ways depending on its location. As a nuclear factor, it interfaces with the p65 subunit of nuclear factor-B (Nf-kB), reducing the expression of proinflammatory genes regulated by it, thus leading to an anti-inflammatory effect [18]. It can also directly bind with chromatin and histones H2A and H2B, stimulate histone deacetylase-3 (HDAC), also regulating gene expression through epigenetic changes in DNA [4]. Differently, upon cellular damage or stress situations, IL-33 reaches the extracellular space in its full-length form, which links to the STL2 membrane-bound receptor, triggering its heterodimerization with IL-1RAcP (IL-1 receptor accessory protein) [19]. Their intracellular domains are in this way connected, resulting in the transduction of IL-33/ST2 signal via different adaptor molecules, such as myeloid differentiation primary response protein 88 (MyD88), IL-1 receptor-associated kinases (IRAK-1 and IRAK-4), and tumor necrosis factor (TNF) acceptor associated factor 6 (TRAF6) [20], which finally bring to the activation of nuclear transcription factors, both directly, as the proinflammatory NF-kB, and with the intermediation of mitogen-activated protein (MAP) kinases p38, c-Jun N-terminal kinase (JNK), and extracellular signal-regulated kinase (ERK) [21]; interestingly, TRAF6 signaling seems to be involved in the pathogenesis of some gastrointestinal cancers [22]. The end results of the IL-33/ST2L signaling pathway are different depending on the ST2L-expressing cell types involved: in epithelial cells, various chemokines are produced, while in TH2 cells, cytokines such as IL-4, IL-5, and IL-13 are released [2]. The activation of different immune cells that follows can shift the cellular components of a TME in a protumorigenic or antitumorigenic direction. Different cellular types are then able to increase IL-33′s bioactivity: neutrophils, abundant in inflammatory environments, secrete cathepsin G, and serine proteases and elastase, which can cleave IL-33 to a ten-fold more powerful isoform; similarly, in allergic reactions, activated mast cells release chymase and tryptase proteases, which cleave IL-33 in thirty-fold more bioactive isoforms [5,23]. Different mechanisms of attenuation of IL-33/ST2 signaling also exist, targeting both IL-33 and ST2L. For example, during apoptosis, IL-33 is inactivated by caspases 3 and 7, preventing its proinflammatory effects if released. The activation of the IL-33/ST2 pathway itself triggers feedback attenuation mechanisms, such as phosphorylation of ST2L by glycogen synthase kinase 3, with its subsequent internalization and degradation by the proteasome following polyubiquitinylation [24]. In the extracellular environment, IL-33 effects are also downregulated by oxidation, transforming its cysteine residues in disulfide bonds [25] and by its binding to the decoy soluble receptor sST2 [2,13,26].

The role of IL-33 in neoplasia has been poorly investigated. The literature shows contrasting results, suggesting that the role of IL-33 in cancer development and growth remains to be clarified. The aim of our review is to summarize the most recent advances in unraveling some of these aspects in gastrointestinal tract cancers, thus highlighting the areas which would benefit from further studies.

## 2. Methods

### 2.1. Search Strategy

The literature search was conducted in the PubMed database (https://pubmed.ncbi.nlm.nih.gov) without restriction on publication period, using the followings terms: (“gastrointestinal cancer”, “colorectal cancer”, “gastric cancer”, “esophageal cancer”) AND (“IL-33”, “Interleukin-33”, “IL-33/ST2 axis”). The “AND” operator was used to create all possible combinations of selected terms.

### 2.2. Study Selection

The initial screening of documents based on abstracts and titles selected only English-language full-text research original articles.

Following the initial Internet search, a total of 92 studies were retrieved from the databases. Thirteen of these were excluded after a review of their titles and abstracts or because they were not available in the English language. Therefore, 79 studies were included in this review.

**Figure 1:**
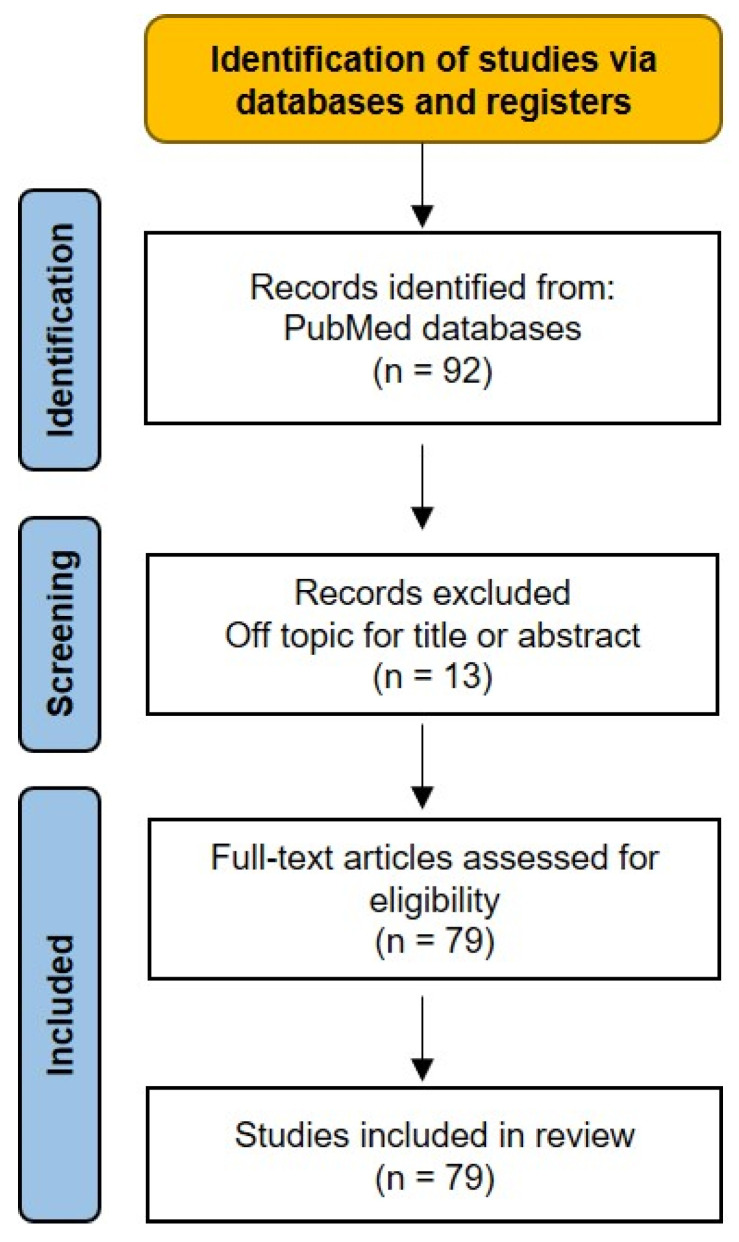
PRISMA 2020 flow diagram for new systematic reviews, which included searches of databases.

The study selection process was performed following the guidelines of Preferred Reporting Items for Systematic Review and Meta-Analysis (PRISMA) [27,28] (Figure 1).

## 3. IL-33 in Gastric Cancer

There were over 1 million new cases of gastric cancer (GC) in 2020 and an estimated 769,000 deaths worldwide, ranking this cancer fifth for incidence and fourth for mortality globally [29]. Rates are two-fold higher in men than in women. Incidence rates are highest in Eastern Asia and Eastern Europe, while rates in North America and Northern Europe are generally low. Recent findings report an increase in the incidence of GC among young adults aged <50 years in both low-risk and high-risk countries, probably due to the surge in autoimmune gastritis prevalence and to the broad diffusion of drugs such as antibiotics and proton pump inhibitors, often tied to gastric dysbiosis [30,31]. Chronic *Helicobacter pylori* infection is considered the principal cause of GC, with the highest prevalence of infection [32,33], but less than 5% of those infected will develop cancer [34]. Established risk factors other than *H. pylori* include Epstein–Barr virus infection, family history, alcohol consumption, tobacco smoking, and dietary factors such as consumption of foods preserved by salting, low fruit intake, and high consumption of processed meat [35,36]. Gastric cancer can anatomically be classified as cardial when developed in proximity to the esophagogastric junction and noncardial, when in the distal portions of the stomach. Furthermore, according to the Lauren classification, there are two histologic subtypes of GC, both associated with *H. pylori*: intestinal adenocarcinoma, characterized by cohesive tumor cells organized in glands and tubules coated by epithelium, mimicking the structure of normal intestinal mucosa, and the diffuse type, which consists of carcinoma cells that lack cohesion and invade tissues independently or in small clusters [37]. The Correa cascade [38] defines the sequence classically thought to lead to GC. The first prolonged precancerous process takes place with chronic active gastritis, chronic atrophic gastritis, and intestinal metaplasia, also known as spasmolytic polypeptide expressing metaplasia (SPEM) when the gastric epithelium is replaced by cells with intestinal phenotype. The final steps are dysplasia with augmented degrees of nuclear polymorphism and irregular architecture, which increases the cancer risk, and finally, invasive carcinoma. IL-33 is constitutively expressed by epithelial cells at the mucosal barrier [39] and also in gastric pit mucous cells and in a small portion of progenitor cells which will differentiate into presurface mucous cells in the normal stomach. IL33 continues to be expressed by surface mucus cells (SMCs) within gastric pits, but it is suppressed as SMCs continue to differentiate and migrate toward the tips of the glands [40]. After parietal cell loss, an increased number of macrophages expressing IL-33 are present within the corpus mucosa [41,42]. IL-33 epithelium-derived “alarmin” can promote a protumorigenic immune response mediated by ST2 receptors on mast cells and via recruitment of immunosuppressive M2 macrophages [39].

### 3.1. In Vivo and In Vitro GC Models

**Figure 2:**
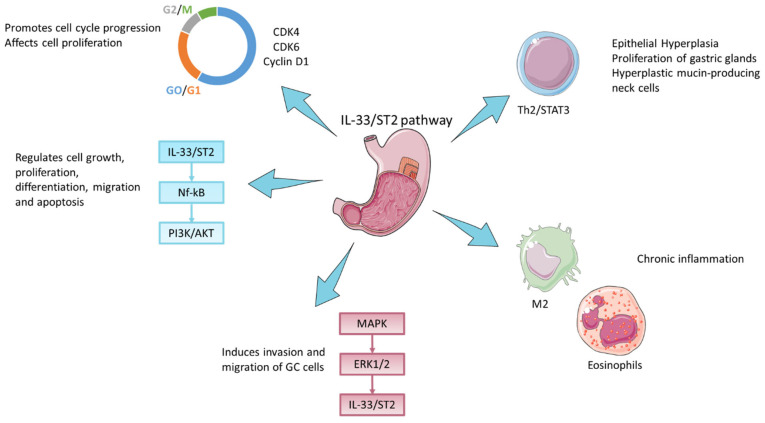
IL-33 in gastric cancer promotes epithelial hyperplasia, which results in the Th2/STAT3-driven proliferation of cells and the appearance of hyperplastic acidic mucin-producing neck cells [40,41]. IL-33 induces M2 macrophage polarization and vigorous infiltration of eosinophils, perpetuating a chronic inflammatory state [41,43]. The IL-33/ST2 axis regulates the expression of cell cycle-associated proteins such as CDK4, CDK6, and cyclin D1 [44] and the expression of pro-proliferative cell cycle genes involved in G0/G1 and G2/M checkpoints [45], thereby promoting cell cycle progression. IL-33/ST2 activates Nf-κB, PI3K/AKT [15,47], and mitogen-activated protein kinases (MAPKs) and ERK1/2 regulating cell growth, proliferation, differentiation, migration, and apoptosis [48,49]. Figure created by Servier Medical Art (smart.servier.com, accessed on 14 March 2023).

Exogenous administration of IL-33 induces SPEM in AKR mice; in fact, the bioactivity of IL-33 promotes epithelial hyperplasia, mainly in goblet cells within GI mucosae, which results in Th2/STAT3-driven gastric pathology [40], the proliferation of cells within the gastric glands, and the appearance of hyperplastic acidic mucin-producing neck cells [41]. IL-33, in addition to being involved in proliferation, apparently when acting directly on the proliferating epithelial cells given their expression of ST2, is also capable of inducing M2 macrophage polarization and vigorous infiltration of eosinophils, perpetuating a chronic inflammatory state that is associated with progression towards a more advanced metaplasia [41,42], and of promoting angiogenesis and tumor cell proliferation [43]. The latter appears to be stimulated by the IL-33/ST2 axis through modulation of the expression of cell cycle-associated proteins, such as CDK4, CDK6, and cyclin D1, resulting in a progression of GC cells along the cell cycle with simultaneous inhibition of apoptosis [44]. It was also reported by Pisani et al., in contrast to the studies above, that IL-33 appears to have a dichotomic role, being antiproliferative and proapoptotic in cancer cell lines while stimulating proliferation and reducing apoptosis in normal epithelial cell lines [45]; these effects may be mediated by the modulation of the expression of pro-proliferative cell cycle genes involved in G0/G1 and G2/M checkpoints [45] (Figure 2). Retrospective studies of human GC have reported that submucosal mast cells in tumor-adjacent tissue promote the growth of GC and participate in the progression of disease and metastasis formation [46]. IL-33 is reported to bind to the ST2 receptor and activate Nf-κB [47], PI3K/AKT [15], and mitogen-activated protein kinases (MAPKs) [48]; the latter can regulate cell growth, proliferation, differentiation, migration, and apoptosis [48] via extracellular signal-regulated kinases, such as ERK1/2 [49] (Figure 1). Consistent with a direct protumorigenic role of IL-33, in *gp130*^F/F^ mice, a murine spontaneous GC model, loss of IL-33 markedly diminishes tumorigenesis and lessens the inflammatory infiltrate, reducing the recruitment of protumorigenic mast cells and M2 macrophages [39].

### 3.2. IL-33 in Human GC

In human tissue samples, IL-33 and ST2 expression is significatively higher in both intestinal metaplasia and GC tissue, compared with control tissue [39]. Furthermore, IL-33 was upregulated in GC patients in comparison with matched normal tissues. Serum levels of IL-33 in patients with GC were significantly higher than in healthy volunteers; moreover, the levels increase with the increase in GC staging from II to III and IV, which suggests that serum IL-33 levels may have a closer correlation with GC development and progression [50]. IL-33 levels in GC patients correlate with several poor prognostic factors, such as depth of invasion, distant metastasis, and advanced stage. Conversely, a recent Chinese study shows lower IL-33 expression levels in GC tissues compared with the adjacent non-neoplastic areas and lower IL-33 circulating levels in GC patients versus healthy controls [51]. These data indicated that IL-33/ST2 is critical for the survival of GC, but its role is not well defined.

Data from the main studies on the role of IL-33 in GC settings are summarized in Table 1.

## 4. IL-33 in Colorectal Cancer

With more than 1.8 million new cases/year in the global population, colorectal cancer (CRC) is the third most common malignancy and the second cause of cancer-related death worldwide, despite important advances in detection, surgery, and chemotherapy [52,53]. Its incidence rates are not homogenous between developing and developed countries, being nearly 4-fold higher in the latter with a 9-fold variation by world region. European regions, Australia, and North America rank the highest; in particular, Hungary and Norway reach the peak incidence, respectively, in male and female populations [29]. A clear genetic predisposition is found in specific syndromes, such as familial adenomatous polyposis and hereditary nonpolyposis colorectal cancer, but only 20% of CRC cases can be linked to them [54]. The largest fraction of CRC cases has been linked to environmental and food-borne mutagens such as heavy alcohol drinking, cigarette smoking, consumption of red or processed meat, specific intestinal commensals and pathogens, and to a sedentary lifestyle with increased prevalence of excess body weight, whereas calcium supplements and adequate consumption of whole grains, fibers, and dairy products appear to be protective factors [55]. Colitis-associated cancer (CAC), the CRC subtype that is associated with inflammatory bowel disease (IBD), is difficult to recognize and treat and has high mortality [56]. IBD patients have a 60% higher risk of CRC compared with the general population [57], and a recent meta-analysis showed that CRC risk in IBD patients rises from 1% to 5% as disease duration increases from 10 to more than 20 years [58].

Some of the essential stages of cancer development are similar between non-inflammatory CRC and CAC. However, different pathogenetic sequences have been proposed for CAC involving chronic inflammation, robust inflammatory infiltration, and increased expression of proinflammatory cytokines [59,60,61]. Expression of IL-33 and its receptor, ST2, positively correlates with the extent of inflammation in IBD patients [10,62].

CRC development can be promoted by fibroblasts, myofibroblasts, epithelial cells, and endothelial cells [63,64,65], in connection with the immune infiltrates in the tumor microenvironment, which modulate the inflammatory milieu in tumor tissues through growth factors and cytokine release [66,67,68]. Recent work indicates that multiple pro-tumorigenic and also anti-tumorigenic cytokines are differently expressed in distinct CRC [69]. The role of IL-33 in intestinal inflammation and CRC development is still unclear. Recent studies have implicated the chronic involvement of the stress response of epithelial cells, which may induce impaired epithelial regeneration [70], and enhanced secretion of inflammatory signals [71], including interleukin (IL)-33. This non-hematopoietic mediated mechanism of IL-33 in the colon impairs the intestinal barrier and may favor microbial translocation that perpetuates colonic inflammation inducing a precancerous setting [62]. Furthermore, an elevated expression of IL-33 was found in tumor tissues in CRC patients, especially in poor-differentiated CRC cells and in genetically altered intestinal epithelial cells, which drive dysplasia [72]. Importantly, these stromal cells regulate the tumor microenvironment to influence CRC initiation and progression and correlate in a dose-dependent manner to promote metastasis formation and progression [73].

### 4.1. In Vivo and In Vitro CRC Models

**Figure 3:**
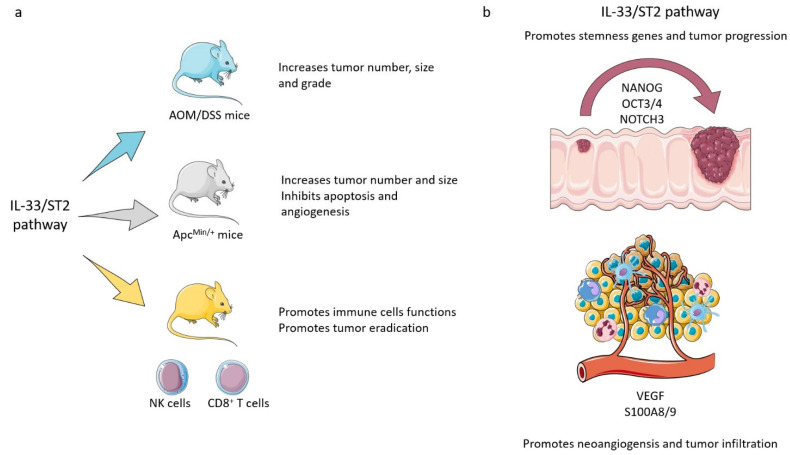
IL-33 in colorectal cancer. (**a**) In the murine models Apc^Min/+^ and AOM/DSS-treated mice, the IL-33/ST2 pathway significantly promotes tumor formation [74,75]. Other studies using in vivo mouse models showed that IL-33 promotes the function of CD8+ T cells and NK cells and, therefore, tumor eradication [76,77]. (**b**) Another possible effect of IL-33/ST2 signaling in human and murine colon cancer cells is the induction of the expression of the core stem cell genes NANOG, NOTCH3, and OCT3/4, enhancing chemoresistance, sphere formation, and in vivo tumor growth [78]. Furthermore, IL-33 stimulates the release of pro-angiogenic factors, such as VEGF and S100A8/9, by macrophages [79]. Figure created by Servier Medical Art (smart.servier.com, accessed on 14 March 2023).

In animal models of colitis, activation of the IL-33/ST2 pathway either inhibits or promotes CRC development [8,41,62]. In azoxymethane (AOM)/DSS-treated mice, the genetic blockade of the IL-33/ST2 pathway significantly prevents tumor formation with a reduction in intestinal tumor number, size, and grade compared with WT mice [74] (Figure 3a). In the Apc^Min/+^ mouse model of intestinal tumorigenesis, genetic and antibody loss of responsiveness to IL-33 reduces tumor number and size by inhibition of proliferation, induction of apoptosis, and suppression of angiogenesis in adenomatous polyps [75] (Figure 3a). These models suggest that the nuclear function of IL-33 as a regulator of gene transcription [4] and its role as a soluble cytokine upon secretion [74] may promote CRC pathogenesis. Other studies using in vivo mouse models showed that IL-33 promotes the function of CD8^+^ T cells and NK cells and, therefore, tumor eradication [76], suggesting that IL-33 signaling may play a protective role against CRC [77]; at the same time, however, its ability to induce cell migration in vitro hints at its involvement in metastasis development in vivo in CRC [73] (Figure 3a).

Another possible effect of IL-33/ST2 signaling is the increase of CRC malignancy mediated by the induction of cancer stem cell-like CRC cells. IL-33/ST2L axis promotes chemoresistance and sphere formation and stimulates in vivo tumor growth, both in human and murine colon cancer cells, with the expression of the core stem cell genes NANOG, NOTCH3, and OCT3/4 [78]. Furthermore, tumor-derived IL-33 is able to recruit macrophages into the tumor microenvironment, where they produce prostaglandin E2, which supports stemness. In addition, IL-33 induces macrophages to release pro-angiogenic factors such as VEGF and S100A8/9 [79] and synergizes with pro-angiogenic factors; this evidence suggests it may promote CRC progression and metastasis [80,81] (Figure 3b).

IL-33 can affect the barrier function of the intestine leading to increased translocation of bacterial products and inducing the production of pro-tumorigenic cytokines, such as IL-6, by immune cells that activate STAT3, thereby promoting tumor growth [74].

### 4.2. IL-33 in Human CRC

Recent studies in CRC patients investigating the role of IL-33/ST2 have shown divergent effects. Several studies observed greater levels of IL-33 and ST2 expression in CRC tissues compared with adjacent normal tissues [73,74,75,79,82], as well as in CRC patients compared with healthy volunteers [75]. Overexpression of both IL-33 and ST2 was reported in intestinal adenomas and adenocarcinomas and is higher in stages I–III low-grade CRC and in stage IV higher-grade and more advanced tumors than in normal tissue [73,82]. The increased expression of the cytokine and its receptor suggests that the IL-33/ST2 axis might play a crucial role in CRC development, eminently in its early stages. As previously reported, tumor localization influences immune response, and CRC patient prognosis [83], and the expression of IL-33 increased in left-sided CRC patients in comparison with right-sided ones, reaching even higher levels in CRC with lymph node (LN) metastasis [84]. It was also noted that the level of desmoplasia, a fibrotic reaction often promoted by cancer-associated fibroblasts and a negative prognostic factor in CRC [85,86], was inversely correlated with stromal ST2 levels, and positively correlated with epithelial IL-33 levels in a group of CRC patients, suggesting a possible role of IL-33/ST2 signaling in desmoplasia development and tumor progression [84]. Data from the main studies on the role of IL-33 in colorectal cancer human and mouse models are summarized in Table 2.

## 5. IL-33 in Esophageal Cancer

There is a small amount of evidence describing the role of IL-33 in other gastrointestinal tract cancers, such as esophageal cancer. This cancer ranks seventh in terms of incidence and sixth in mortality overall [29]. The burden is heavier on male individuals, which represent 70% of total cases, and higher rates are observed in developed than in developing countries for men, while there is no significative difference considering females [87]. Esophageal cancer incidence substantially differs between the two most common histologic subtypes: squamous cell carcinoma (ESCC), for which the major risk factors are heavy drinking and smoking and their synergistic effects, and adenocarcinoma (EAC), favored by an excess of body weight, gastroesophageal reflux disease (GERD), and Barrett’s esophagus [87].

### 5.1. In Vivo ESCC and EAC Models

**Figure 4:**
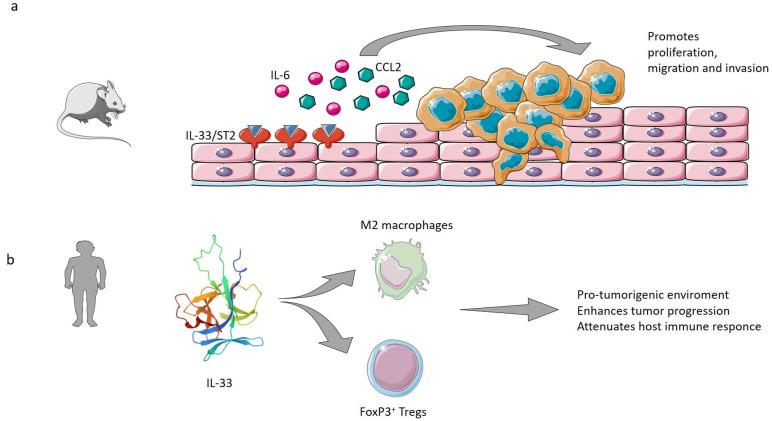
IL-33 in esophageal cancer. (**a**) In mouse models, the expression of metastasis-related molecules, such as CCL2, and proinflammatory cytokines, such as IL-6 [88] was upregulated by IL-33, indicating its ability to promote proliferation, migration, and invasion of esophageal adenocarcinoma cells through ST2 [85,86,87]. (**b**) In humans, IL-33 increases the number of polarized M2 macrophages and increases the density of stromal FoxP3+ Tregs, which are thought to enhance tumor progression attenuating the host immune response [93,94,95]. Figure created by Servier Medical Art (www.smart.servier.com, accessed on 14 March 2023). IL-33 structure obtained from Protein Data Bank (https://www.rcsb.org/structure/2kll, accessed on 14 March 2023; PDB DOI: 10.2210/pdb2KLL/pdb).

In mouse models, the expression of metastasis-related molecules, such as CCL2, was upregulated by IL-33, indicating its ability to promote invasion and migration in ESCC as well as in other cancers [88,89,90]. During the progression of GERD to EAC, IL-33, likely behaving as an alarmin responding to acidic insult, was unwaveringly elevated and localized into the cytoplasm of epithelial cells, from where it enacts its pro-proliferative effects and stimulates migration and invasion of tumor cells through ST2, while simultaneously inducing secretion of IL-6 [91] (Figure 4a). In a rat model of gastroesophageal reflux, which simulates the progression from normal to low-grade dysplasia, high-grade dysplasia, and EAC, IL-33 increased gradually, suggesting its involvement in the entire process from esophageal inflammation to tumorigenesis of EAC [91].

### 5.2. IL-33 in Human ESCC

More attention has been directed in the literature toward the role of IL-33 in ESCC. Higher levels of IL-33 have been found in the tumor tissues of ESCC patients than in adjacent normal tissues, also appearing closely related to ESCC progression, invasive depth, degree of differentiation, TNM stage, and worse clinical outcomes [88], although without a clear correlation with overall survival. Furthermore, higher levels of IL-33 in ESCC tissues have also been correlated with the concomitant increased numbers of M2 macrophages: the cytokine proves to be able to promote M2 polarization via the ornithine decarboxylase (ODC) enzyme, favoring a pro-tumorigenic environment [92]. A correlation has also been observed in ESCC tissues between higher levels of IL-33 and increased density of stromal FoxP3+ Tregs [93], which are thought to enhance tumor progression attenuating the host immune response against ESCC [94,95] (Figure 4b).

Table 3 summarizes data from the main studies on the role of IL-33 in esophageal cancer in human and rodent models.

## 6. Conclusions

From this review of the literature, it is evident that the IL-33/ST2 axis plays a complex, multifaceted role in the carcinogenesis of gastrointestinal tract tumors. Parallels can be observed between the effects of its activation in esophageal, stomach, and colorectal cancers. It works as an orchestrator of the composition of the tumor microenvironment, activating or inhibiting various cellular types, mostly of the immune family, but also directly on the cancerous cells themselves. Its final role, either pro- or antitumorigenic, appears to be ambivalent, probably varying in relation to the different phases or types of the inflammatory and carcinogenic process or to other factors yet to be identified. In fact, it has been shown to be involved both in the inflammatory process leading to degeneration and neoplasia and also in the characterization of cancer itself with stem cell-like, angiogenetic, and metastatic properties. A more precise definition of the roles of the IL-33/ST2 axis in each different step of the tumorigenic process has yet to be reached; such a definition is necessary to better understand if the axis can be targeted to obtain regression or prevention of tumorigenesis.

## Figures and Tables

**Table 1:** Summary of the main studies on the role of IL-33 in human and mice gastric cancer models. GES-1: human gastric epithelial cells; MGC-803: human gastric carcinoma cells; MKN45, AGS: human gastric adenocarcinoma; NCI-N87: human gastric carcinoma cell line; SGC-7901, BGC823: human gastric cancer cell line; SPEM: spasmolytic polypeptide-expressing metaplasia.

Article	Experimental System	Proposed Mechanism of Action of IL-33/ST2	Role of IL-33/ST2
Buzzelli et al., 2015 [40]	Rag-1^−/−^ mouse model; human biopsy specimens	Protection against Th1-biased immune response and subsequent precancerous progression	Antitumorigenic
Yu et al., 2015 [49]	Human gastric cancer cell lines MGC-803, BGC-823, and SGC-7901	Promotion of invasion and migration of GC cells via the ST2/ERK1/2 pathway, through the MAPK pathway	Protumorigenic
Petersen et al., 2017 [42]	Murine models with SPEM (DMP-777-treated) or advanced SPEM (L635-treated)	Metaplasia induction and macrophage polarization to M2	Protumorigenic
Eissmann et al., 2019 [43]	Human gastric cancer tissue samples; murine models	Mast cell recruitment of tumor-associated macrophages via the gastric cancer cell-derived IL-33/ST2 axis and promotion of tumor cell proliferation and angiogenesis	Protumorigenic
De Salvo et al., 2020 [41]	SAMP1/YitFc (SAMP) gastritis-prone murine model	Promotion of gastritis SPEM through recruitment of eosinophils and IL33-expressing M2 macrophages	Protumorigenic
Huang et al., 2021 [44]	GC cell lines AGS and MKN45	Proliferation and cell cycle progression of GC cells, upregulation of CDK4, CDK6, and cyclin D1; inhibition of apoptosis and stimulation of invasion and migration of GC cells	Protumorigenic
Pisani et al., 2021 [45]	GES-1, AGS, and NCI-N87 human cell lines; ex vivo human gastric cancer tissue samples	Antiproliferative and proapoptotic effect on cancer cell lines, and it can stimulate proliferation and reduce apoptosis in normal epithelial cell lines	Dichotomic
Tran et al., 2022 [39]	Human gastric tissue samples; murine models *Tff1*^−/−^, *gp130*^F/F^, *Il33*^−/−^	Recruitment of protumorigenic mast cells and M2 macrophages	Protumorigenic

**Table 2:** Summary of the main studies on the role of IL-33 in human and mice colorectal cancer models. AOM: azoxymethane; CAF: cancer-associated fibroblast; CRC: colorectal cancer; CT26, MC38: murine colorectal carcinoma cell line; CXCR4: C-X-C chemokine receptor type 4; DSS: dextran sulfate sodium; HCT116, RKO, COLO205, HCT115, LoVo, MOSER, SW620, and SW480: human colon carcinoma cell lines; HT29, Caco2: human colorectal adenocarcinoma cell lines; IL-6: interleukin-6; JKN: c-Jun N-terminal kinases; MMP2 and MMP9: matrix metallopeptidase 2 and 9; NANOG: homeobox protein NANOG; NOTCH3: notch receptor 3; OCT3/4: octamer-binding transcription factor 4; PGE2: prostaglandin E2.

Article	Experimental System	Proposed Mechanism of Action of IL-33/ST2	Role of IL-33/ST2
Oboki et al., 2010 [8]	Murine models of DSS-induced colitis	Stimulates both local inflammation via neutrophil-chemoattractant factors, and resolution of tissue damage during DSS-induced ‘innate’ colitis	Dichotomic
Yang et al., 2011 [76]	CD4-CRE Eomes fl/fl/Tbet doubly deficient mice, CD4-cre Eomes fl/fl mice, and Pmel-1 TCR transgenic mice; mice lymphocyte CD8^+^ T cells culture	Stimulation of T-CD8^+^ and NK cells’ antitumoral functions	Antitumorigenic
Sedhom et al., 2013 [62]	Mice deficient for St2 (St2^−/−^) and for IL-33 (Il33^−/−^); human Caco-2 cell line	Impairment of intestinal epithelial barrier function and subsequent translocation of bacteria stimulating inflammation	Protumorigenic
Liu et al., 2014 [73]	Human SW620 cells	Promotion of tumoral growth and metastasis through increased expression of IL-6, CXCR4, MMP2, and MMP9	Protumorigenic
Maywald et al., 2015 [75]	Apc^Min/+^ mouse model of intestinal tumorigenesis	Stimulation of proliferation and angiogenesis, and inhibition of apoptosis in adenomatous polyps, through stimulation of myofibroblasts and mast cells in tumor microenvironment	Protumorigenic
Cui et al., 2015 [82]	Human CRC and colonic adenomatous tissue samples	Regulation of angiogenesis	Protumorigenic
Mertz et al., 2016 [74]	Resected human CRC specimens; murine CRC models AOM/DSS treated	Impairment of the intestinal barrier integrity and triggering the production of pro-tumorigenic IL-6 by immune cells	Protumorigenic
O’Donnell et al., 2016 [77]	Human CRC samples; CT26 cells engraftment on BALB/c mice.	Promotion of macrophage and CD8^+^ T cell infiltration	Antitumorigenic
Fang et al., 2017 [78]	Human CCR tissue specimens; human HT-29 cell line; murine MC38 cell line	Enhancement of in vivo tumor growth and chemoresistance through expression of the core stem cell genes NANOG, NOTCH3, and OCT3/4 and activation of JNK. Recruitment of PGE2 producing macrophages	Protumorigenic
Zhang et al., 2017 [79]	Murine CT26 and MC38 cell lines; murine models of tumor cell engraftments; human HCT116, HT29, Caco2, RKO, COLO205, HCT115, LoVo, MOSER, and SW480 cell lines	Stimulation of tumoral angiogenesis and metastasis	Protumorigenic
Landskron et al., 2019 [84]	HT29 and HCT116 cell lines; CRC tissue samples	CAF-mediated promotion of invasion and metastasis by activating desmoplasia	Protumorigenic

**Table 3:** Summary of the main studies about the role of IL-33 in human and rodent esophageal cancer models. ESCC: Esophageal squamous cell carcinoma; EAC: esophageal adenocarcinoma; CCL2: chemokine (C-C motif) ligand 2; KYSE-450: human esophageal squamous carcinoma cell line; Eca-109, OE19, OE33: human esophageal carcinoma cells; HEEC: human esophageal epithelial cell.

Article	Experimental System	Proposed Mechanism of Action of IL-33/ST2	Role of IL-33/ST2
Cui G et al. 2019 [93]	ESCC human tissue samples	Recruitment of Tregs.	Protumorigenic
Yue Y et al. 2020 [88]	KYSE-450 and Eca-109 esophageal cancer cells	Promotion of ESCC tumor development and metastasis by recruiting regulatory T cells (Tregs) through CCL2	Protumorigenic
Mai S et al. 2021 [92]	ECA109 esophageal cancer cells; Tumor xenograft in mice.	Induction of M2-like macrophage polarization in ESCC tumor microenvironment	Protumorigenic
Liu J et al. 2022 [91]	Esophageal adenocarcinoma cells (OE19 and OE33) and human esophageal epithelial cells (HEECs); EAC rat model	Enhancement of proliferation, migration, invasion, and epithelial-mesenchymal transition (EMT) in EAC cells.	Protumorigenic

## Data Availability

Not applicable.

## References

[1] Baekkevold E.S., Roussigné M., Yamanaka T., Johansen F.E., Jahnsen F.L., Amalric F., Brandtzaeg P., Erard M., Haraldsen G., Girard J.P. (2003). Molecular characterization of NF-HEV, a nuclear factor preferentially expressed in human high endothelial venules. Am. J. Pathol..

[2] Schmitz J., Owyang A., Oldham E., Song Y., Murphy E., McClanahan T.K., Zurawski G., Moshrefi M., Qin J., Li X. (2005). IL-33, an interleukin-1-like cytokine that signals via the IL-1 receptor-related protein ST2 and induces T helper type 2-associated cytokines. Immunity.

[3] Fagundes C.T., Amaral F.A., Souza A.L., Vieira A.T., Xu D., Liew F.Y., Souza D.G., Teixeira M.M. (2007). ST2, an IL-1R family member, attenuates inflammation and lethality after intestinal ischemia and reperfusion. J. Leukoc. Biol..

[4] Carriere V., Roussel L., Ortega N., Lacorre D.A., Americh L., Aguilar L., Bouche G., Girard J.P. (2007). IL-33, the IL-1-like cytokine ligand for ST2 receptor, is a chromatin-associated nuclear factor in vivo. Proc. Natl. Acad. Sci. USA.

[5] Lefrançais E., Roga S., Gautier V., Gonzalez-de-Peredo A., Monsarrat B., Girard J.P., Cayrol C. (2012). IL-33 is processed into mature bioactive forms by neutrophil elastase and cathepsin G. Proc. Natl. Acad. Sci. USA.

[6] Lefrançais E., Cayrol C. (2012). Mechanisms of IL-33 processing and secretion: Differences and similarities between IL-1 family members. Eur. Cytokine Netw..

[7] Liew F.Y., Pitman N.I., McInnes I.B. (2010). Disease-associated functions of IL-33: The new kid in the IL-1 family. Nat. Rev. Immunol..

[8] Oboki K., Ohno T., Kajiwara N., Arae K., Morita H., Ishii A., Nambu A., Abe T., Kiyonari H., Matsumoto K. (2010). IL-33 is a crucial amplifier of innate rather than acquired immunity. Proc. Natl. Acad. Sci. USA.

[9] Miller A.M. (2011). Role of IL-33 in inflammation and disease. J. Inflamm..

[10] Pastorelli L., Garg R.R., Hoang S.B., Spina L., Mattioli B., Scarpa M., Fiocchi C., Vecchi M., Pizarro T.T. (2010). Epithelial-derived IL-33 and its receptor ST2 are dysregulated in ulcerative colitis and in experimental Th1/Th2 driven enteritis. Proc. Natl. Acad. Sci. USA.

[11] Bejarano L., Jordāo M.J.C., Joyce J.A. (2021). Therapeutic Targeting of the Tumor Microenvironment. Cancer Discov..

[12] Leibovici J., Itzhaki O., Huszar M., Sinai J. (2011). The tumor microenvironment: Part 1. Immunotherapy.

[13] Larsen K.M., Minaya M.K., Vaish V., Peña M.M.O. (2018). The Role of IL-33/ST2 Pathway in Tumorigenesis. Int. J. Mol. Sci..

[14] Jovanovic I., Radosavljevic G., Mitrovic M., Juranic V.L., McKenzie A.N., Arsenijevic N., Jonjic S., Lukic M.L. (2011). ST2 deletion enhances innate and acquired immunity to murine mammary carcinoma. Eur. J. Immunol..

[15] Jovanovic I.P., Pejnovic N.N., Radosavljevic G.D., Arsenijevic N.N., Lukic M.L. (2012). IL-33/ST2 axis in innate and acquired immunity to tumors. Oncoimmunology.

[16] Zerdes I., Matikas A., Foukakis T. (2023). The interplay between eosinophils and T cells in breast cancer immunotherapy. Mol. Oncol..

[17] Blomberg O.S., Spagnuolo L., Garner H., Voorwerk L., Isaeva O.I., van Dyk E., Bakker N., Chalabi M., Klaver C., Duijst M. (2023). IL-5-producing CD4(+) T cells and eosinophils cooperate to enhance response to immune checkpoint blockade in breast cancer. Cancer Cell.

[18] Ali S., Mohs A., Thomas M., Klare J., Ross R., Schmitz M.L., Martin M.U. (2011). The dual function cytokine IL-33 interacts with the transcription factor NF-kappaB to dampen NF-kappaB-stimulated gene transcription. J. Immunol..

[19] Zhao W., Hu Z. (2010). The enigmatic processing and secretion of interleukin-33. Cell Mol. Immunol..

[20] Funakoshi-Tago M., Tago K., Hayakawa M., Tominaga S., Ohshio T., Sonoda Y., Kasahara T. (2008). TRAF6 is a critical signal transducer in IL-33 signaling pathway. Cell Signal.

[21] Liew F.Y., Girard J.P., Turnquist H.R. (2016). Interleukin-33 in health and disease. Nat. Rev. Immunol..

[22] Bradley J.R., Pober J.S. (2001). Tumor necrosis factor receptor-associated factors (TRAFs). Oncogene.

[23] Lefrancais E., Duval A., Mirey E., Roga S., Espinosa E., Cayrol C., Girard J.P. (2014). Central domain of IL-33 is cleaved by mast cell proteases for potent activation of group-2 innate lymphoid cells. Proc. Natl. Acad. Sci. USA.

[24] Zhao J., Wei J., Mialki R.K., Mallampalli D.F., Chen B.B., Coon T., Zou C., Mallampalli R.K., Zhao Y. (2012). F-box protein FBXL19-mediated ubiquitination and degradation of the receptor for IL-33 limits pulmonary inflammation. Nat. Immunol..

[25] Cohen E.S., Scott I.C., Majithiya J.B., Rapley L., Kemp B.P., England E., Rees D.G., Overed-Sayer C.L., Woods J., Bond N.J. (2015). Oxidation of the alarmin IL-33 regulates ST2-dependent inflammation. Nat. Commun..

[26] Hayakawa H., Hayakawa M., Kume A., Tominaga S. (2007). Soluble ST2 blocks interleukin-33 signaling in allergic airway inflammation. J. Biol. Chem..

[27] Page M.J., McKenzie J.E., Bossuyt P.M., Boutron I., Hoffmann T.C., Mulrow C.D., Shamseer L., Tetzlaff J.M., Akl E.A., Brennan S.E. (2021). The PRISMA 2020 statement: An updated guideline for reporting systematic reviews. BMJ.

[28] Page M.J., Moher D., Bossuyt P.M., Boutron I., Hoffmann T.C., Mulrow C.D., Shamseer L., Tetzlaff J.M., Akl E.A., Brennan S.E. (2021). PRISMA 2020 explanation and elaboration: Updated guidance and exemplars for reporting systematic reviews. BMJ.

[29] Sung H., Ferlay J., Siegel R.L., Laversanne M., Soerjomataram I., Jemal A., Bray F. (2021). Global Cancer Statistics 2020: GLOBOCAN Estimates of Incidence and Mortality Worldwide for 36 Cancers in 185 Countries. CA Cancer J. Clin..

[30] Anderson W.F., Rabkin C.S., Turner N., Fraumeni J.F., Rosenberg P.S., Camargo M.C. (2018). The Changing Face of Noncardia Gastric Cancer Incidence Among US Non-Hispanic Whites. J. Natl. Cancer Inst..

[31] Camargo M.C., Anderson W.F., King J.B., Correa P., Thomas C.C., Rosenberg P.S., Eheman C.R., Rabkin C.S. (2011). Divergent trends for gastric cancer incidence by anatomical subsite in US adults. Gut.

[32] Plummer M., Franceschi S., Vignat J., Forman D., de Martel C. (2015). Global burden of gastric cancer attributable to Helicobacter pylori. Int. J. Cancer.

[33] Hooi J.K.Y., Lai W.Y., Ng W.K., Suen M.M.Y., Underwood F.E., Tanyingoh D., Malfertheiner P., Graham D.Y., Wong V.W.S., Wu J.C.Y. (2017). Global Prevalence of Helicobacter pylori Infection: Systematic Review and Meta-Analysis. Gastroenterology.

[34] Kidd M., Lastovica A.J., Atherton J.C., Louw J.A. (1999). Heterogeneity in the Helicobacter pylori vacA and cagA genes: Association with gastroduodenal disease in South Africa?. Gut.

[35] Nagini S. (2012). Carcinoma of the stomach: A review of epidemiology, pathogenesis, molecular genetics and chemoprevention. World J. Gastrointest. Oncol..

[36] Wasmer M.H., Krebs P. (2016). The Role of IL-33-Dependent Inflammation in the Tumor Microenvironment. Front. Immunol..

[37] Thrift A.P., Nguyen T.H. (2021). Gastric Cancer Epidemiology. Gastrointest. Endosc. Clin. N. Am..

[38] Correa P. (2013). Gastric cancer: Overview. Gastroenterol. Clin. N. Am..

[39] Tran C.P., Scurr M., O’Connor L., Buzzelli J.N., Ng G.Z., Chin S.C.N., Stamp L.A., Minamoto T., Giraud A.S., Judd L.M. (2022). IL-33 promotes gastric tumour growth in concert with activation and recruitment of inflammatory myeloid cells. Oncotarget.

[40] Buzzelli J.N., Chalinor H.V., Pavlic D.I., Sutton P., Menheniott T.R., Giraud A.S., Judd L.M. (2015). IL33 Is a Stomach Alarmin That Initiates a Skewed Th2 Response to Injury and Infection. Cell Mol. Gastroenterol. Hepatol..

[41] De Salvo C., Pastorelli L., Petersen C.P., Buttò L.F., Buela K.A., Omenetti S., Locovei S.A., Ray S., Friedman H.R., Duijser J. (2020). IL-33 triggers early eosinophil-dependent events leading to metaplasia in a chronic model of gastritis-prone mice. Gastroenterology.

[42] Petersen C.P., Meyer A.R., De Salvo C., Choi E., Schlegel C., Petersen A., Engevik A.C., Prasad N., Levy S.E., Peebles R.S. (2018). A signalling cascade of IL-33 to IL-13 regulates metaplasia in the mouse stomach. Gut.

[43] Eissmann M.F., Dijkstra C., Jarnicki A., Phesse T., Brunnberg J., Poh A.R., Etemadi N., Tsantikos E., Thiem S., Huntington N.D. (2019). IL-33-mediated mast cell activation promotes gastric cancer through macrophage mobilization. Nat. Commun..

[44] Huang N., Cui X., Li W., Zhang C., Liu L., Li J. (2021). IL-33/ST2 promotes the malignant progression of gastric cancer via the MAPK pathway. Mol. Med. Rep..

[45] Pisani L.F., Tontini G.E., Gentile C., Marinoni B., Teani I., Nandi N., Creo P., Asti E., Bonavina L., Vecchi M. (2021). Proinflammatory Interleukin-33 Induces Dichotomic Effects on Cell Proliferation in Normal Gastric Epithelium and Gastric Cancer. Int. J. Mol. Sci..

[46] Zhao Y., Wu K., Cai K., Zhai R., Tao K., Wang G., Wang J. (2012). Increased numbers of gastric-infiltrating mast cells and regulatory T cells are associated with tumor stage in gastric adenocarcinoma patients. Oncol. Lett..

[47] Wu Y., Wang F., Fan L., Zhang W., Wang T., Du Y., Bai X. (2018). Baicalin alleviates atherosclerosis by relieving oxidative stress and inflammatory responses via inactivating the NF-κB and p38 MAPK signaling pathways. Biomed. Pharmacother..

[48] Sui X., Kong N., Ye L., Han W., Zhou J., Zhang Q., He C., Pan H. (2014). p38 and JNK MAPK pathways control the balance of apoptosis and autophagy in response to chemotherapeutic agents. Cancer Lett..

[49] Yu X.X., Hu Z., Shen X., Dong L.Y., Zhou W.Z., Hu W.H. (2015). IL-33 Promotes Gastric Cancer Cell Invasion and Migration Via ST2-ERK1/2 Pathway. Dig. Dis. Sci..

[50] Sun P., Ben Q., Tu S., Dong W., Qi X., Wu Y. (2011). Serum interleukin-33 levels in patients with gastric cancer. Dig. Dis. Sci..

[51] Liu Q.H., Zhang J.W., Xia L., Wise S.G., Hambly B.D., Tao K., Bao S.S. (2022). Clinical implications of interleukins-31, 32, and 33 in gastric cancer. World J. Gastrointest. Oncol..

[52] Baidoun F., Elshiwy K., Elkeraie Y., Merjaneh Z., Khoudari G., Sarmini M.T., Gad M., Al-Husseini M., Saad A. (2021). Colorectal Cancer Epidemiology: Recent Trends and Impact on Outcomes. Curr. Drug. Targets.

[53] Wong M.C.S., Huang J., Lok V., Wang J., Fung F., Ding H., Zheng Z.J. (2021). Differences in Incidence and Mortality Trends of Colorectal Cancer Worldwide Based on Sex, Age, and Anatomic Location. Clin. Gastroenterol. Hepatol..

[54] Rustgi A.K. (2007). The genetics of hereditary colon cancer. Genes Dev..

[55] Siegel R.L., Miller K.D., Fuchs H.E., Jemal A. (2022). Cancer statistics, 2022. CA Cancer J. Clin..

[56] Feagins L.A., Souza R.F., Spechler S.J. (2009). Carcinogenesis in IBD: Potential targets for the prevention of colorectal cancer. Nat. Rev. Gastroenterol. Hepatol..

[57] Lutgens M.W., van Oijen M.G., van der Heijden G.J., Vleggaar F.P., Siersema P.D., Oldenburg B. (2013). Declining risk of colorectal cancer in inflammatory bowel disease: An updated meta-analysis of population-based cohort studies. Inflamm. Bowel Dis..

[58] Herrinton L.J., Liu L., Levin T.R., Allison J.E., Lewis J.D., Velayos F. (2012). Incidence and mortality of colorectal adenocarcinoma in persons with inflammatory bowel disease from 1998 to 2010. Gastroenterology.

[59] Neurath M.F. (2014). Cytokines in inflammatory bowel disease. Nat. Rev. Immunol..

[60] Waldner M.J., Neurath M.F. (2008). Cytokines in colitis associated cancer: Potential drug targets?. Inflamm. Allergy Drug Targets.

[61] Atreya I., Neurath M.F. (2008). Immune cells in colorectal cancer: Prognostic relevance and therapeutic strategies. Expert Rev. Anticancer Ther..

[62] Sedhom M.A., Pichery M., Murdoch J.R., Foligné B., Ortega N., Normand S., Mertz K., Sanmugalingam D., Brault L., Grandjean T. (2013). Neutralisation of the interleukin-33/ST2 pathway ameliorates experimental colitis through enhancement of mucosal healing in mice. Gut.

[63] Calon A., Espinet E., Palomo-Ponce S., Tauriello D.V., Iglesias M., Céspedes M.V., Sevillano M., Nadal C., Jung P., Zhang X.H. (2012). Dependency of colorectal cancer on a TGF-β-driven program in stromal cells for metastasis initiation. Cancer Cell.

[64] Lu J., Ye X., Fan F., Xia L., Bhattacharya R., Bellister S., Tozzi F., Sceusi E., Zhou Y., Tachibana I. (2013). Endothelial cells promote the colorectal cancer stem cell phenotype through a soluble form of Jagged-1. Cancer Cell.

[65] Calon A., Lonardo E., Berenguer-Llergo A., Espinet E., Hernando-Momblona X., Iglesias M., Sevillano M., Palomo-Ponce S., Tauriello D.V., Byrom D. (2015). Stromal gene expression defines poor-prognosis subtypes in colorectal cancer. Nat. Genet..

[66] Kantola T., Klintrup K., Väyrynen J.P., Vornanen J., Bloigu R., Karhu T., Herzig K.H., Näpänkangas J., Mäkelä J., Karttunen T.J. (2012). Stage-dependent alterations of the serum cytokine pattern in colorectal carcinoma. Br. J. Cancer.

[67] Krzystek-Korpacka M., Diakowska D., Kapturkiewicz B., Bębenek M., Gamian A. (2013). Profiles of circulating inflammatory cytokines in colorectal cancer (CRC), high cancer risk conditions, and health are distinct. Possible implications for CRC screening and surveillance. Cancer Lett..

[68] De Simone V., Pallone F., Monteleone G., Stolfi C. (2013). Role of T. Oncoimmunology.

[69] Mlecnik B., Bindea G., Angell H.K., Sasso M.S., Obenauf A.C., Fredriksen T., Lafontaine L., Bilocq A.M., Kirilovsky A., Tosolini M. (2014). Functional network pipeline reveals genetic determinants associated with in situ lymphocyte proliferation and survival of cancer patients. Sci. Transl. Med..

[70] Kaser A., Blumberg R.S. (2011). Autophagy, microbial sensing, endoplasmic reticulum stress, and epithelial function in inflammatory bowel disease. Gastroenterology.

[71] Strober W., Fuss I.J. (2011). Proinflammatory cytokines in the pathogenesis of inflammatory bowel diseases. Gastroenterology.

[72] Terzic J., Grivennikov S., Karin E., Karin M. (2010). Inflammation and colon cancer. Gastroenterology.

[73] Liu X., Zhu L., Lu X., Bian H., Wu X., Yang W., Qin Q. (2014). IL-33/ST2 pathway contributes to metastasis of human colorectal cancer. Biochem. Biophys. Res. Commun..

[74] Mertz K.D., Mager L.F., Wasmer M.H., Thiesler T., Koelzer V.H., Ruzzante G., Joller S., Murdoch J.R., Brümmendorf T., Genitsch V. (2016). The IL-33/ST2 pathway contributes to intestinal tumorigenesis in humans and mice. Oncoimmunology.

[75] Maywald R.L., Doerner S.K., Pastorelli L., De Salvo C., Benton S.M., Dawson E.P., Lanza D.G., Berger N.A., Markowitz S.D., Lenz H.J. (2015). IL-33 activates tumor stroma to promote intestinal polyposis. Proc. Natl. Acad. Sci. USA.

[76] Yang Q., Li G., Zhu Y., Liu L., Chen E., Turnquist H., Zhang X., Finn O.J., Chen X., Lu B. (2011). IL-33 synergizes with TCR and IL-12 signaling to promote the effector function of CD8+ T cells. Eur. J. Immunol..

[77] O’Donnell C., Mahmoud A., Keane J., Murphy C., White D., Carey S., O’Riordain M., Bennett M.W., Brint E., Houston A. (2016). An antitumorigenic role for the IL-33 receptor, ST2L, in colon cancer. Br. J. Cancer.

[78] Fang M., Li Y., Huang K., Qi S., Zhang J., Zgodzinski W., Majewski M., Wallner G., Gozdz S., Macek P. (2017). IL33 Promotes Colon Cancer Cell Stemness via JNK Activation and Macrophage Recruitment. Cancer Res..

[79] Zhang Y., Davis C., Shah S., Hughes D., Ryan J.C., Altomare D., Peña M.M. (2017). IL-33 promotes growth and liver metastasis of colorectal cancer in mice by remodeling the tumor microenvironment and inducing angiogenesis. Mol. Carcinog..

[80] Choi Y.S., Choi H.J., Min J.K., Pyun B.J., Maeng Y.S., Park H., Kim J., Kim Y.M., Kwon Y.G. (2009). Interleukin-33 induces angiogenesis and vascular permeability through ST2/TRAF6-mediated endothelial nitric oxide production. Blood.

[81] Stojkovic S., Kaun C., Heinz M., Krychtiuk K.A., Rauscher S., Lemberger C.E., de Martin R., Gröger M., Petzelbauer P., Huk I. (2014). Interleukin-33 induces urokinase in human endothelial cells--possible impact on angiogenesis. J. Thromb. Haemost..

[82] Cui G., Qi H., Gundersen M.D., Yang H., Christiansen I., Sørbye S.W., Goll R., Florholmen J. (2015). Dynamics of the IL-33/ST2 network in the progression of human colorectal adenoma to sporadic colorectal cancer. Cancer Immunol. Immunother..

[83] Lee G.H., Malietzis G., Askari A., Bernardo D., Al-Hassi H.O., Clark S.K. (2015). Is right-sided colon cancer different to left-sided colorectal cancer?—A systematic review. Eur. J. Surg. Oncol..

[84] Landskron G., De la Fuente López M., Dubois-Camacho K., Díaz-Jiménez D., Orellana-Serradell O., Romero D., Sepúlveda S.A., Salazar C., Parada-Venegas D., Quera R. (2019). Interleukin 33/ST2 Axis Components Are Associated to Desmoplasia, a Metastasis-Related Factor in Colorectal Cancer. Front. Immunol..

[85] Ueno H., Shinto E., Shimazaki H., Kajiwara Y., Sueyama T., Yamamoto J., Hase K. (2015). Histologic categorization of desmoplastic reaction: Its relevance to the colorectal cancer microenvironment and prognosis. Ann. Surg. Oncol..

[86] Ueno H., Kanemitsu Y., Sekine S., Ishiguro M., Ito E., Hashiguchi Y., Kondo F., Shimazaki H., Mochizuki S., Kajiwara Y. (2017). Desmoplastic Pattern at the Tumor Front Defines Poor-prognosis Subtypes of Colorectal Cancer. Am. J. Surg. Pathol..

[87] Arnold M., Abnet C.C., Neale R.E., Vignat J., Giovannucci E.L., McGlynn K.A., Bray F. (2020). Global Burden of 5 Major Types of Gastrointestinal Cancer. Gastroenterology.

[88] Yue Y., Lian J., Wang T., Luo C., Yuan Y., Qin G., Zhang B., Zhang Y. (2020). Interleukin-33-nuclear factor-κB-CCL2 signaling pathway promotes progression of esophageal squamous cell carcinoma by directing regulatory T cells. Cancer Sci..

[89] Moisan F., Francisco E.B., Brozovic A., Duran G.E., Wang Y.C., Chaturvedi S., Seetharam S., Snyder L.A., Doshi P., Sikic B.I. (2014). Enhancement of paclitaxel and carboplatin therapies by CCL2 blockade in ovarian cancers. Mol. Oncol..

[90] Zhang J., Lu Y., Pienta K.J. (2010). Multiple roles of chemokine (C-C motif) ligand 2 in promoting prostate cancer growth. J. Natl. Cancer Inst..

[91] Liu J., Liu L., Su Y., Wang Y., Zhu Y., Sun X., Guo Y., Shan J. (2022). IL-33 Participates in the Development of Esophageal Adenocarcinoma. Pathol. Oncol. Res..

[92] Mai S., Liu L., Jiang J., Ren P., Diao D., Wang H., Cai K. (2021). Oesophageal squamous cell carcinoma-associated IL-33 rewires macrophage polarization towards M2 via activating ornithine decarboxylase. Cell Prolif..

[93] Cui G., Li Z., Ren J., Yuan A. (2019). IL-33 in the tumor microenvironment is associated with the accumulation of FoxP3-positive regulatory T cells in human esophageal carcinomas. Virchows Arch..

[94] Nabeki B., Ishigami S., Uchikado Y., Sasaki K., Kita Y., Okumura H., Arigami T., Kijima Y., Kurahara H., Maemura K. (2015). Interleukin-32 expression and Treg infiltration in esophageal squamous cell carcinoma. Anticancer Res..

[95] Vacchelli E., Semeraro M., Adam J., Dartigues P., Zitvogel L., Kroemer G. (2016). Immunosurveillance in esophageal carcinoma: The decisive impact of regulatory T cells. Oncoimmunology.

